# Learning from Decoys to Improve the Sensitivity and Specificity of Proteomics Database Search Results

**DOI:** 10.1371/journal.pone.0050651

**Published:** 2012-11-26

**Authors:** Amit Kumar Yadav, Dhirendra Kumar, Debasis Dash

**Affiliations:** GNR Knowledge Center for Genome Informatics, CSIR-Institute of Genomics and Integrative Biology, Delhi, India; UGent/VIB, Belgium

## Abstract

The statistical validation of database search results is a complex issue in bottom-up proteomics. The correct and incorrect peptide spectrum match (PSM) scores overlap significantly, making an accurate assessment of true peptide matches challenging. Since the complete separation between the true and false hits is practically never achieved, there is need for better methods and rescoring algorithms to improve upon the primary database search results. Here we describe the calibration and False Discovery Rate (FDR) estimation of database search scores through a dynamic FDR calculation method, FlexiFDR, which increases both the sensitivity and specificity of search results. Modelling a simple linear regression on the decoy hits for different charge states, the method maximized the number of true positives and reduced the number of false negatives in several standard datasets of varying complexity (18-mix, 49-mix, 200-mix) and few complex datasets (E. coli and Yeast) obtained from a wide variety of MS platforms. The net positive gain for correct spectral and peptide identifications was up to 14.81% and 6.2% respectively. The approach is applicable to different search methodologies- separate as well as concatenated database search, high mass accuracy, and semi-tryptic and modification searches. FlexiFDR was also applied to Mascot results and showed better performance than before. We have shown that appropriate threshold learnt from decoys, can be very effective in improving the database search results. FlexiFDR adapts itself to different instruments, data types and MS platforms. It learns from the decoy hits and sets a flexible threshold that automatically aligns itself to the underlying variables of data quality and size.

## Introduction

Database searching is an important step in high-throughput proteomics analysis and requires computational tools that can assign spectra with good statistical confidence. Due to an inherent lack of complete fragmentation knowledge it is difficult to separate the interesting spectra (containing peptide sequence information) from the uninteresting noisy ones. Controlling an expected proportion of false positives above a threshold is a useful and preferred methodology [1], known as the false discovery rate (FDR) [2]. FDR has become a widely accepted method for multiple testing corrections in genomics [3] and proteomics [1]. Search engines will invariably score all searched spectra. Some spectra do not originate from peptides while the correct peptides for others are absent in the database searched. These spectra are assigned to incorrect peptide sequences. Such PSMs need to be discriminated for better automated survey of the proteomes. In an ideal scenario, a mass spectrometry instrument should spew out perfect data (without noise, contaminants or any other systemic aberrations). This goes into a hypothetical perfect search engine that identifies all proteins present and does not identify anything else. In reality, raw database search scores need to be calibrated for better discrimination between target and decoy hits which is an important but difficult task in post database search workflows. In general, applying filters on the search results is a popular method of post processing [4]–[6]. For example, XCorr and ΔCn for Sequest [7]–[11], Mascot identity and homology thresholds [12], [13], e-value based filters for X!Tandem [14] and OMSSA [15]. The issue has started to be taken more seriously and many algorithms have been devised to tackle the peptide identification quality [16] from primary database search results. The quality control of peptide matching is a matter of high concern [17] and employing robust statistics based on target-decoy strategy and other statistical models for re-scoring and FDR calculation have been of help in many studies. Methods based on machine learning have been developed for better information retrieval from the mass spectrometry data [18]–[21]. For example- Peptide Prophet which was developed originally for SEQUEST [22], and was further improved by exploiting the target decoy strategy [23], [24]. Percolator [12], [25], [26] and PROVALT [13] used decoy for enhancing Sequest and Mascot performance. Apart from these, there are many other tools that have been interfaced with the common search algorithms to enhance the number and quality of matches using machine learning and statistical techniques like linear discriminant function [10], [24], [27], non-linear discriminant function [28], clustering [29], regression [9] and Bayesian models [30], [31]. Non-linear curve fitting has also been used in the PSPEP method employed in Protein Pilot for calculating local FDRs [32].

**Figure 1:**
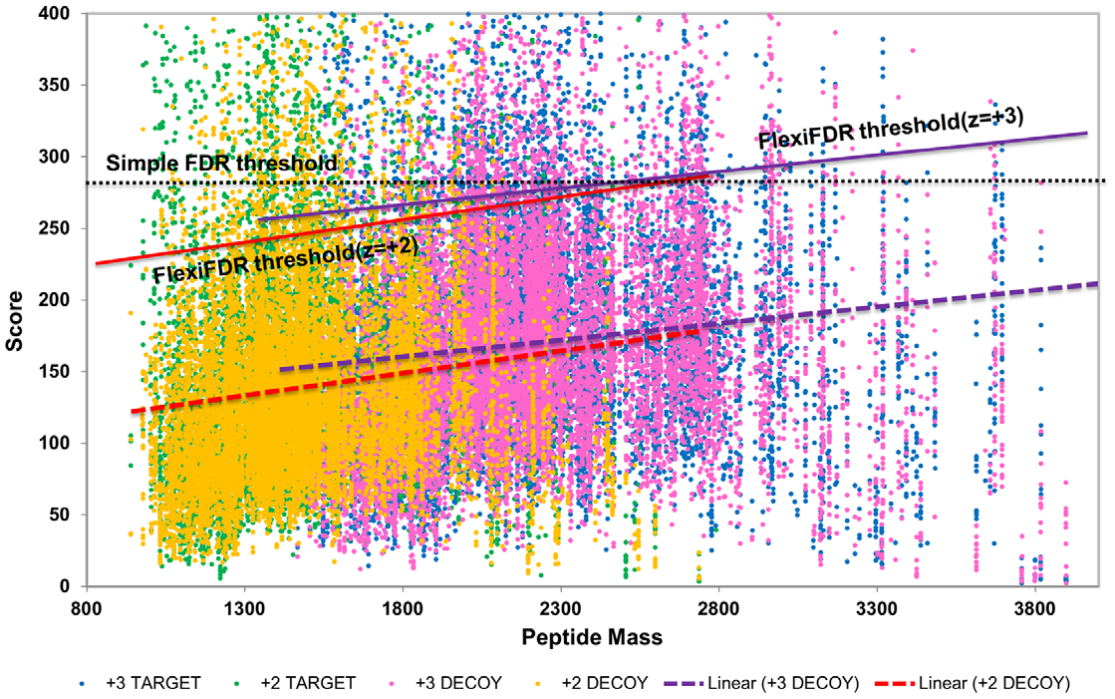
The concept of FlexiFDR method. The linear regression line of decoy hits is represented by the line equation 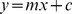 to show the effect of increasing mass on decoy hits (PSMs) in QTOF dataset. Two lines are shown for two different charge states (+2 and +3). When a simple FDR is calculated on MassWiz scores (shown by dotted line), many correct hits (green and blue dots) are lost in lower mass regions with high density. The FlexiFDR method uses a line 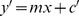 for every charge state (colored solid lines), parallel to the decoy line of that charge, as a dynamic threshold based on decoy results to estimate FDR. The scores are transformed using this equation of line. This method helps in enhancing the true hits and decreasing the false hits at <1%FDR and reduces the time spent for manual validation.

Our method is aimed at utilizing the information content from decoy results for increasing the sensitivity and specificity of database search results taking MassWiz [33] search algorithm as an example. Its applicability is also demonstrated for Mascot. MassWiz is an open-source algorithm which performs with high discriminative power like Mascot. It has been a part of two large scale studies –mining the affinity-depleted plasma proteome [34] and Mycobacterium tuberculosis H37Rv proteogenomics [35]. Improving its performance will be helpful to the community as an open-source alternative to proprietary search tools. Most machine learning methods take database search scores and other related features for discriminant analysis. The resulting coefficients may not be generally applicable to different datasets and different platforms. A better alternative is to have platform specific scoring system with known features obviating the need for discriminant analysis. Nevertheless, no scoring system can be perfect and some discriminative power is contained in features other than the raw scores [36] (like ΔCn, peptide length/mass, shared peak counts etc.). Our method tries to account for bias caused by correlated variables as examined from a decoy search.

Utilizing the decoy database as a null model, we explored the decoy results to gain insights into MassWiz and Mascot score properties, understand the inherent weaknesses and improve the results, if possible. MassWiz is based on peptide fragmentation heuristics that include product ion continuity, intensities, supporting neutral losses and immonium ions customized for different mass spectrometric(MS)-platforms, imparting it good discriminative power. This has one shortcoming- as the peptide mass increases, so does the scores. This results in neglecting true hits from low mass region and accepting false hits from high mass regions. Similar but opposite effect is observed for Mascot scores. The degree of this effect is variable for various charge states and also for data sets from different MS-platforms. Therefore, proposed methods for score normalization and re-calibration (as in case of XCorr) did not work. Setting different thresholds for different mass regions using mass-bin based approaches [37] can be used to exploit the bias. Although this is a good strategy, a noticeable drawback is the requirement of a wider mass tolerance search which is time-consuming. We tackle the problem with a regression based method. Taking advantage of the mass bias of decoys, we use a linear regression model on mass and score for different charge states to calculate the average decoy score for any given mass and based on this regression line, the threshold is set as a parallel line that can be adjusted according to the desired FDR level. This is highly adaptive to different data-sets, instrument types and search parameters and thus directly learns the bias from decoy search results. It is not dependent on any specific type of search strategy and works well on both separate and concatenated search strategies, ppm tolerance searches, semi-tryptic searches and searches with variable modifications.

**Figure 2:**
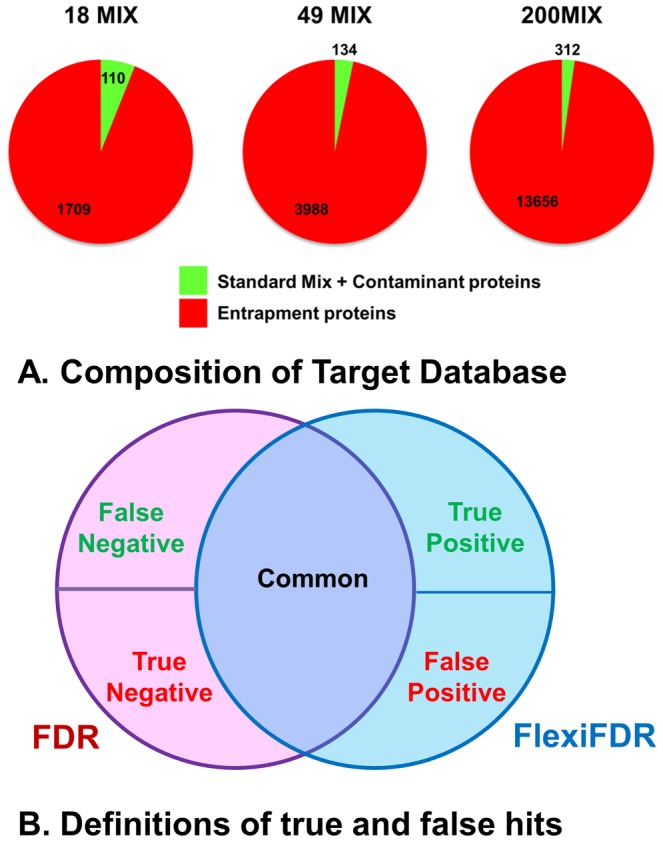
Database composition and evaluation terminology. (A) The composition of databases used for searching standard mix datasets is shown. Database consists of standard mix proteins and common contaminants, both of which are considered true proteins (shown in green). It also consists of sequences from an unrelated organism which represent the entrapment sequences or false proteins (shown in red). The sizes of these two parts show that the true proteins were outnumbered by entrapment sequences. (B) For evaluating the FlexiFDR method, the definitions of true and false positives and negatives are relative to the unique sets identified by only one method- either FDR or FlexiFDR.

**Figure 3:**
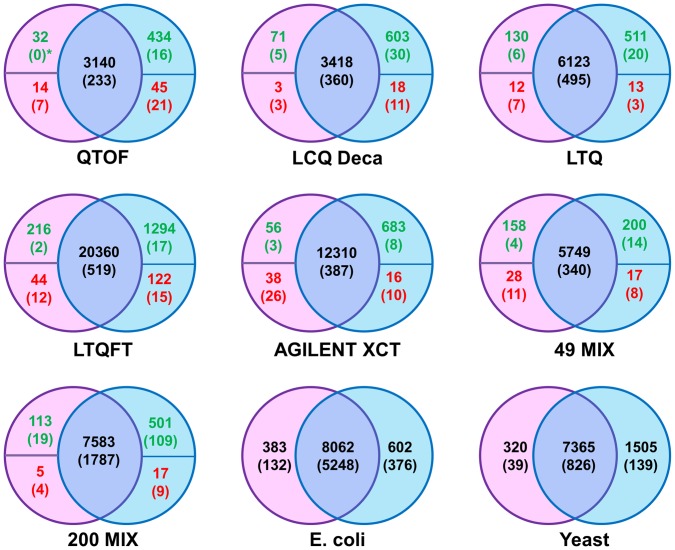
Comparison of spectra and peptides assigned by FDR and FlexiFDR for concatenated search. Comparison of spectra and peptides assigned by FDR (pink) and FlexiFDR (blue) for concatenated database search. The number of spectra is shown on top with the number peptides in brackets beneath them. For the standard mixtures, the true positives (green) and false positives (red) identified exclusively are highlighted. FlexiFDR identifies a higher number of true unique spectra and peptides than FDR in almost all cases. The proportion of false positives in exclusively identified set is higher in FDR than FlexiFDR. A star symbol (*) depicts that although there are non-zero true positive spectra identifications in few cases of FDR, they could not bring in any new peptide identification. The peptides they identified were already identified by other spectra (which are shared by both FDR and FlexiFDR).

## Results and Discussion

The MassWiz scores were found to be correlated with peptide mass. With an increase in mass, the decoy scores increased and this effect was seen to be affected by charge state (Figure 1). For higher charge states (∼5 or more), the mass dependence may weaken or show negative effect. This effect was also observed for data sets from various platforms and few of them have been shown in Figure S1. By regressing decoy hits based on charge, a variable FDR threshold could be calculated for different peptide masses. This method, named FlexiFDR, was applied to various diverse data sets. To evaluate the accuracy of FlexiFDR, we tested it across instruments, data types, MS platforms and search methodology. For accomplishing this, we used known standard mixtures of increasing complexity-18,49 and 200 mix, obtained from disparate instrument types and calculated the FDR using both separate and concatenated database search strategies. A strict FDR threshold of ≤1% was applied to all search results before comparison. After calculating FDR with general and FlexiFDR method, comparisons were made at unique spectra and peptide levels. All results provided in main text are compiled from concatenated search results while the separate search results are provided as supplementary figures.

**Figure 4:**
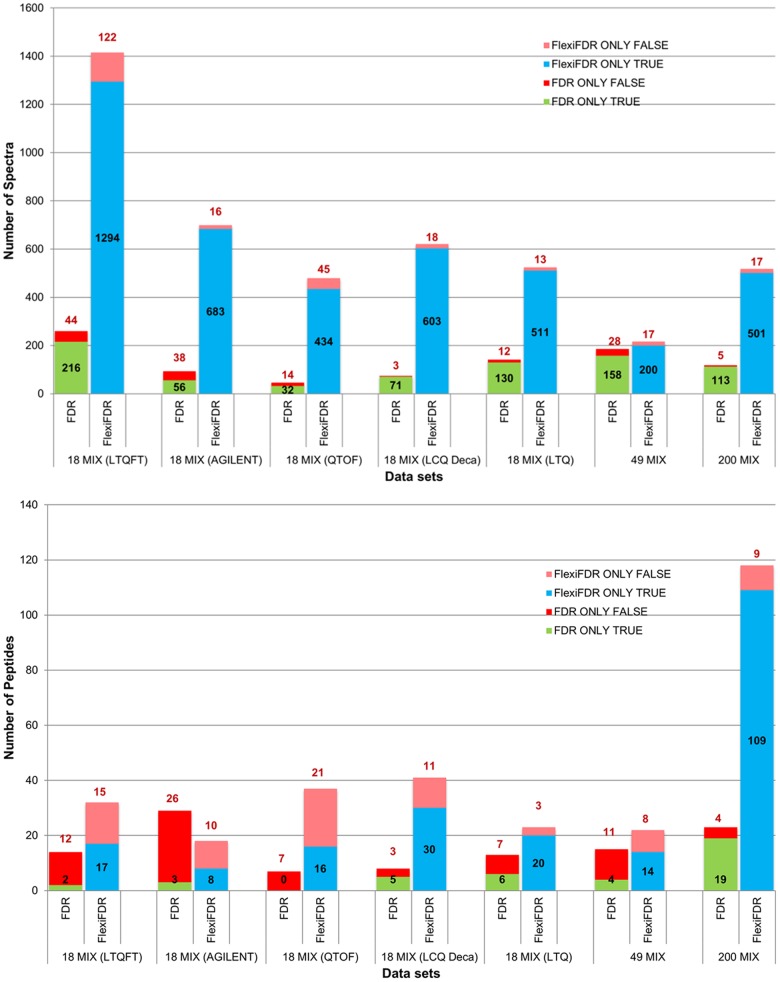
Comparison of unique identifications (spectra and peptides) from concatenated search. Top and Bottom panels depict spectra and corresponding peptide comparison from a concatenated search. The blue colored bars represent the unique true hits added by FlexiFDR alone while green colored bars represent unique true hits from FDR alone. Similarly, the pink bars denote false hits from FlexiFDR alone while red bars denote false hits from FDR alone. The spectral hits from FDR can be mapped to unique peptides right in the lower panel. The false spectral hits in case of FDR alone bring more false peptide identifications than FlexiFDR (compare bars from A to B vertically). FlexiFDR brings more unique true hits than FDR and brings lesser number of unique false hits. This enhances the true positives and decreases false positives in the datasets shown.

For comparative evaluation, the related terminology is explained in Figure 2. Since FlexiFDR primarily is a rescoring method, most PSM and peptide identifications compared to general FDR are expected to be common. Comparing the number of hits (PSMs and peptides) does not provide a true picture. The true positives, false positives, true negatives and false negatives are defined with respect to FlexiFDR (Figure 2). Several datasets of varying complexity were searched with both separate and concatenated database search approaches, called FDR_s_ and FDR_c_ respectively. The comparisons for concatenated search are shown as Venn diagrams in Figure 3. Similar results are observed for separate database search (Figure S2). The complete results are tabulated as Table S1 and S2.

**Figure 5:**
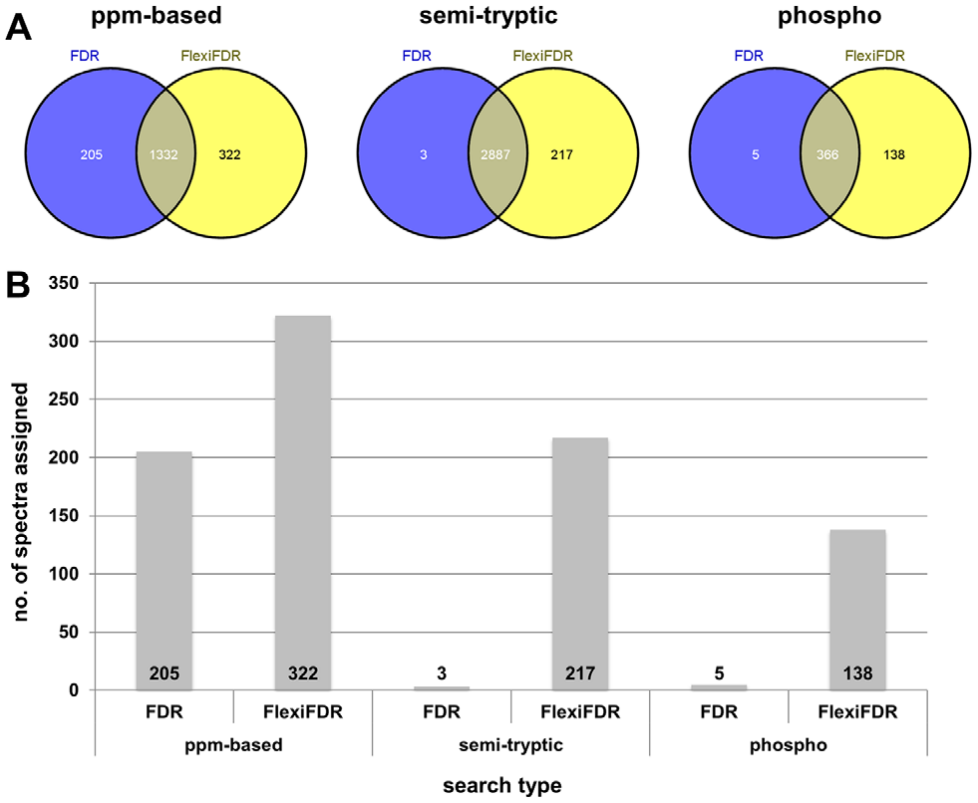
ppm, semi-tryptic and modification searches. This figure denotes versatility of FlexiFDR on ppm based (plasma data), semi tryptic (QTOF) and Phosphorylation modification searches (Phospho data). Details for searches are given in methods. These different searches depict improved performance after applying FlexiFDR. Panel A shows direct Venn comparisons for the three searches. Their corresponding unique spectra are compared in bar graphs below them in panel B to show the effect.

**Figure 6:**
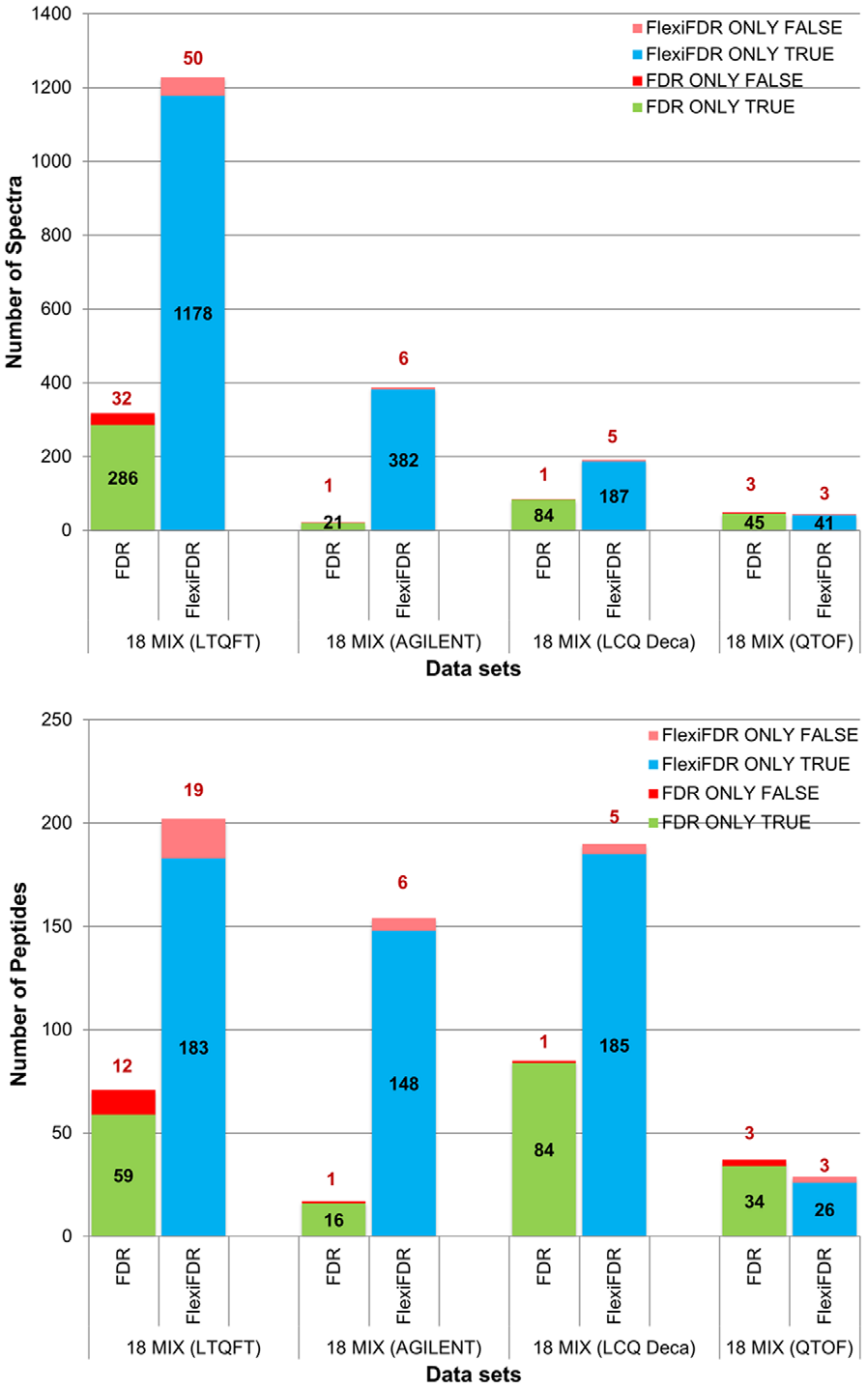
FlexiFDR on Mascot concatenated searches. FlexiFDR was also applied to Mascot and results for concatenated searches are shown for the unique identifications in some standard mix datasets. Except for QTOF, the other datasets showed improvement in number of spectra and peptide identifications.

Analysis of identifications unique to FDR and FlexiFDR provides a better depiction of the merit of one method over the other. A comparison of the unique identifications from FDR_c_ for standard data sets is represented as bar graphs in Figure 4. Figure S3 depicts similar results for comparison of separate searches. FlexiFDR leads to higher number of unique identifications in both methods. The numbers of true identifications are much higher in FlexiFDR as compared to FDR. FlexiFDR also decreases the false positives thereby enhancing the performance (Figures 3 and 4). FlexiFDR could enhance up to 14.81% Net Positive Gain in spectra identifications and upto 6.2% peptide identifications (Table S1). On an average, FlexiFDR identified up to ∼ 4.33% net positive gains in spectral identifications and 3.55% in peptide identifications in the standard mix datasets (Table S1.A and S1.B). For unique identifications, the net positive gain was up to ∼13.85 times more true spectral hits and up to ∼2.3 times more true peptide hits (Table S1 and S2).

**Table 1:** Terminology used for assessing the quality of database peptide matches to spectra.

Term	Definitions (also see Fig 2A and 2B)
True Positive	All identified matches (PSMs/Peptides) at 1% FDR that come from a standard protein or a known contaminant and found only in FlexiFDR but not simple FDR
False Positive	All identified matches (PSMs/Peptides) at 1% FDR that come from unrelated/entrapment organism (*H. influenza/Mycobacterium tuberculosis/Rhodobacter sphaeroides*/*E.coli*) proteins, are not shared by standard proteins or known contaminants, and are found only in FlexiFDR but not simple FDR
True Negative	All identified matches (PSMs/Peptides) at 1% FDR that come from *unrelated/entrapment organisms’ proteins(mentioned above)*, are not shared by standard proteins or known contaminants, and are identified only by simple FDR but not FlexiFDR
False Negative	All identified matches at 1% FDR that correspond to the standard mix proteins and identified contaminants, and are found only in simple FDR but not FlexiFDR

In general, it is known that lower mass peptides have a greater chance of being a false positive. By lowering the threshold in low mass region, one should expect more false positives. However, we have shown that proper threshold learnt from decoys, can be very effective in improving the results even at lower mass regions. Employing a charge based threshold allows for flexible modeling irrespective of the slope of the linear regression.

For the complex data sets from E. coli and Yeast, since the true and false identifications cannot be easily defined, we compared their identifications by showing number of spectral and peptide identifications (Figure 3 and Figure S2). The comparisons at 1%FDR threshold are tabulated in Table S2. We observed that FlexiFDR assigned more spectra and peptides for both FDR_s_ and FDR_c_. Average Percentage gain in spectral identification was 8.29% and peptide identification was 7.05%. Unique identifications were enhanced by more than double increment in spectra and peptide numbers.To check whether the trends hold true for different kinds of searches, we carried out high mass accuracy searches (ppm level), searches with semitryptic option and searches with variable modifications of phosphorylation at serine, threonine and tyrosine residues. In all these searches, similar trends were observed and FlexiFDR application resulted in better performance (Figure 5). The Venn diagrams (Figure 5A) and Bar graphs (Figure 5B) show that FlexiFDR is applicable across different methods of data analysis.

To further explore the mass dependency, we tried to observe the effect on different search algorithms. We found that X!Tandem and OMSSA being dependent on calibrated e-values, do not have such bias. Interestingly, X!Tandem’s raw score, the hyper score, shows such a dependence (Figure S4). Mascot ion score, however, showed negative dependence on mass (Figure S5). Since FlexiFDR depends on the slope, it can adapt to any linear relation with mass and charge. FlexiFDR was applied to few standard datasets for Mascot for evaluation. As expected, we found better results (Figure 6) except for QTOF dataset where the results were nearly similar to previous results. These results show that this method is applicable to other algorithms as well and is versatile in application.

### Conclusion

This approach noticeably has many advantages- it adapts itself to different instruments, data types and MS platforms. Given any dataset, it learns from the decoys and sets a flexible threshold that automatically aligns itself to the underlying variables of data quality and size. It recovers many border line true spectra. By recovering true spectra and eliminating false ones, this method will aid in improved performance in label-free quantitation studies. It is also easily applicable to other algorithms after the correlated variables have been found. Although we have shown charge and mass dependence in this work, it could be other variables for different algorithms.

The slopes of decoy regression lines shown in this study are positive. But FlexiFDR is not restricted to work only on such data. It will work even if different charge states have different slopes including a mixture of positive and negative slopes for different charges. This has been successfully applied on Mascot results depicting its broader utility. For higher charge state data (>5), sometimes there are low number of spectra acquired. This may not be suitable for this method if the points are too few and skewed towards one side. Large datasets should benefit more from FlexiFDR method. Another related pitfall is that FlexiFDR might not work on very small datasets since it needs enough data to learn accurately from it. This is a general property of any FDR method per se and therefore it cannot be used where FDR cannot. The method is simple to use and extensible in design. It can be freely downloaded from https://sourceforge.net/projects/mssuite/files/FlexiFDR/.

## Materials and Methods

### Data sets and Database Search

Several standard mixtures of increasing complexity (18 mix, 49 mix and 200 mix) along with few complex data sets of Yeast and E. coli from high resolution instruments were used to demonstrate the enhancement due to FlexiFDR. Additionally, one dataset from our previous study [34] (A14S3) was used to demonstrate effect of ppm searches. A human phosphorylated data set [38] was used to depict the effect of variable modification (phosphorylation).

The MS/MS spectra (MIX 3) were taken from 18 protein mixture [39] Seattle Proteome Center (http://regis-web.systemsbiology.net/PublicDatasets). ISB standard mix as described in Klimek et al. [40] was downloaded and searched as described in MassWiz article. MzXML files from the Mix 3 dataset were converted to mascot generic format (mgf) using mzXML2Search program. The mgf files for each instrument type were collated into a single mgf file for search. The target database consisted of standard mix proteins (true), known contaminants (true) and appended H. influenza proteins (entrapment or false).

A standard 49-mix dataset downloaded from peptidome. (PSE 108) converted to mgf and collated together. This was searched against a database of 49 proteins with contaminants (true) and an appended database of Mycobacterium tuberculosis H37Rv (entrapment or false). This dataset was searched with the following parameters-trypsin enzyme with 1 missed cleavage, fixed modification of Carbamidomethylation, variable modification of Methionine oxidation, peptide tolerance 1 Da and fragment tolerance 0.8 Da.

A complex standard mix of 200 proteins, SC 200 (Seattle Children 200), developed by Bauman et al. [41] (kindly provided by Dr. Eugene Kolker, personal communication). Database used for search was constituted from the standard proteins (true), common contaminants (true) from cRAP (112 sequences from http://www.thegpm.org/crap/index.html), unrelated organisms (entrapment or false) -*Rhodobacter sphaeroides* (4131 sequences) and *E.coli* (9525 sequences). Precursor ion tolerance of 1 Da, product ion tolerance of 0.6 Da, trypsin digestion with 1 missed cleavage, a fixed modification of +57.03Da (Carbamidomethylation) at Cysteine residues.

Mid log phase Yeast dataset [42] PSM 1001 (from PSE 101) downloaded from peptidome. For the yeast data set from ESI-TRAP, a 3 Da error window was allowed for precursors while fragment masses were allowed to be matched at 0.6 Da. Tryptic digestion with 1 missed cleavage was considered with carbamidomethylation as the fixed modification and oxidation of methionine residues as variable modification for the search.

E. coli dataset [43] PSM 1224 (from PSE 126) downloaded from peptidome. This data was searched with 30 ppm precursor tolerance and 0.5 Da fragment tolerance, instrument type-FTICR, trypsin enzyme with 1 missed cleavage, fixed modification of carbamidomethylation at cysteine residues, variable modifications of deamidation and methionine oxidation.

Semi tryptic search in MassWiz was carried out for QTOF dataset with similar parameters as above except for semi-tryptic cleavage. MassWiz search for Phosphorylated dataset [38] was conducted in Human protein database (RefSeq) with 20 ppm precursor accuracy and 0.8 Da fragment tolerance, trypsin with 2 missed cleavages. Carbamidomethylation was defined as fixed modification and Phosphorylation of STY residues as variable modification along with methionine oxidation. One dataset from our previous study on plasma [34], A14S1 was used to depict effect of ppm search. The database searches were performed with 10 ppm precursor and 0.6 Da fragment ion tolerances in IPI Human database (v3.74). All cysteines were considered modified with carbamidomethylation and a variable modification of methionine oxidation was also taken into account. Tryptic digestion with a maximum of 2 missed cleavages was considered.

Effect of mass on X!Tandem hyperscore was observed on QTOF dataset searched with following parameters - 2Da precursor tolerance, 0.6 Da fragment tolerance, trypsin with one missed cleavage, fixed modification of carbamidomethyl and methionine oxidation as variable modification.

For analysis and validation of the robustness of an algorithm/analysis pipeline, a gold standard dataset is an important pre-requisite. A protein mixture with known proteins (and well known contaminants) can effectively act as a standard dataset. Several attempts at providing such standard datasets have advanced the computational proteomics field [40], [41], [44]–[46]. There is no assurance that all ionized peptides from these proteins (along with the known contaminants) can be identified or all peptides identified from these proteins are essentially correct. But, at strict False Discovery Rates (FDR) ≤1% used throughout this study, it can be safely assumed that the PSMs from standard samples can be considered as true hits. Recently, Granholm et al. [47] assessed the statistical calibration of scores using samples of known proteins and entrapment sequences. Borrowing their terminology, the proteins other than those from the standard mix and known contaminants will be referred to as entrapment sequences i.e. known incorrect proteins from target database which can be used to assess the actual FDR but not directly used for FDR calculation. For estimation of FDR, a decoy database was created by reversing all target database proteins. The terminology used is described in Table 1 and Figure 2. This terminology aids in objective comparison and assessment of the performance of the new algorithm introduced in this paper.

### False Discovery Rate Calculation

All searches were initially conducted as separate target-decoy searches. FDR for both separate target-decoy method [48] and concatenated target-decoy method [1] was calculated from the same files using the ProteoStats library (developed in house). The decoy peptides which had an identical peptide in target database were ignored from decoy results during FDR calculation. Leu/Ile were considered indistinguishable and treated as identical. FDR was calculated from database search scores. The FDR for separate target-decoy search, FDR_s_, was calculated as -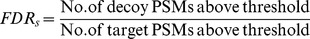
and the FDR for concatenated target-decoy search, FDR_c_ was calculated as -



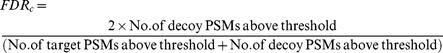



The target and decoy scores were sorted in descending order and FDR calculated at each decoy score taken as the threshold. The score at which the FDR was calculated to be 1% or immediately below 1% (i.e. FDR ≤1%) was taken as the score threshold.

### FlexiFDR Methodology

Better separation of target and decoy results is an important aspect of current proteomics research. Decoy results from multiple search results were explored to understand the reasons for false positives and negatives. MassWiz decoy scores were observed to be dependent on peptide mass and charge state. Performing a linear regression on the decoy hits based on mass for different charge states provides a better alternative for FDR calculation. A bin based approach could help but that does not provide fine control while a linear regression gives a smooth threshold. It can be considered akin to an infinitely small bins approach to calculate FDR. For rescoring the results for better discrimination, the decoy scores were fit using a linear regression model against the peptide mass. This is an indirect effect caused due to peptide length and charge which are known to cause differential fragmentation in Collision induced dissociation (CID) [49]. There is no directly predictable rule which can be modelled into any scoring function per se. This effect is highly variable for peptide length, instrument type, collision energy etc. As shown in Figure 1, if the peptide mass effect is unknown, we calculate FDR on the scores using a fixed threshold on y-axis. In the generally followed simple FDR scenario, it is a linear threshold across all peptide masses (shown by dotted line). The FlexiFDR threshold is a dynamic threshold set according to the score distribution of decoy results for different charge states. This threshold is a line parallel to the decoy regression line (shown by dashed line for different charges). Application of this threshold for different charges helps accentuate many true positives (see Table 1 for terminology), i.e. matches that were originating from correct proteins and removing many false positives. Many correct PSMs that were on the borderline region (just below FDR) could now be assigned and some incorrect PSMs that earlier passed the threshold, could now be removed at the same FDR threshold (≤1%FDR). This approach improved the sensitivity and specificity of the algorithm.

For implementation of FlexiFDR algorithm, the linear regression of decoy hits was modelled as an equation of a line, which provides an analytical function to adjudge the mean decoy scores at a particular mass.

In other words, using this analytical function, one can predict what would be the average random hit score for any given mass. But this is of little use directly since we are not interested in knowing the average decoy score.

By drawing a line parallel to this decoy regression line, flexible threshold for FDR can be calculated for different charge states.

Before the regression, all peptides from decoy that resembled target peptides were removed. Leu and Ile were considered as indistinguishable and thus were considered identical. Linear regression is then performed for different charge states by taking mass as independent variable and score as dependent one. After the regression line is calculated and the slope m is determined, we can calculate a parallel line through every point (with coordinate- mass, score) that gives a projection (in the form of intercept) on the y-axis. For every decoy and target score as y’, and known slope m, we calculate the intercept c’ that becomes the new score.
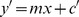



This is easy to calculate from the above equation. The next step calculates FDR using this new score, called FlexiScore. In effect, this rescoring brings about the desired flexible threshold using an analytical algebraic function, which in essence gives the score’s projection on y-axis after learning the trend from decoy hits. The advantage of this method is the ease of calculation, robustness and accuracy.

## Supporting Information

Figure S1 **Mass bias trends for few more datasets.** The figure shows the mass bias trend as shown for QTOF data in figure 1. This depicts the observation of the mass bias trend for different charge states in few more datasets. This observation is repeatable and forms the basis of FlexiFDR.
(TIF)

Figure S2 **Comparison of spectra and peptides assigned by FDR and FlexiFDR for separate search.** Comparison of spectra and peptides assigned by FDR (pink) and FlexiFDR (blue) for separate database search. The number of spectra is shown on top with the number peptides in brackets beneath them. For the standard mixtures, the true positives (green) and false positives (red) identified exclusively are highlighted. FlexiFDR identifies a higher number of true unique spectra and peptides than FDR in almost all cases. The proportion of false positives in exclusively identified set is higher in FDR than FlexiFDR. A star symbol (*) depicts that although there are non-zero true positive spectra identifications in few cases of FDR, they could not bring in any new peptide identification. The peptides they identified were already identified by other spectra (which are shared by both FDR and FlexiFDR).
(TIF)

Figure S3 **Comparison of unique identifications (spectra and peptides) from separate search.** Top and Bottom panels depict spectra and corresponding peptide comparison from a separate search. The blue colored bars represent the unique true hits added by FlexiFDR alone while green colored bars represent unique true hits from FDR alone. Similarly, the pink bars denote false hits from FlexiFDR alone while red bars denote false hits from FDR alone. The spectral hits from FDR can be mapped to unique peptides right in the lower panel. The false spectral hits in case of FDR alone bring more false peptide identifications than FlexiFDR (compare bars from A to B vertically). FlexiFDR brings more unique true hits than FDR and brings lesser number of unique false hits. This enhances the true positives and decreases false positives in the datasets shown. The FlexiFDR method is not search strategy dependent.
(TIF)

Figure S4 **Mass bias trend for X!Tandem hyperscore.** Although X!Tandem values do not show mass bias, the hyperscore does show dependence on mass and thus the same trend as MassWiz. QTOF dataset is shown here as an example.
(TIF)

Figure S5 **Mass bias trend for Mascot ion score.** Mascot ion scores also show a mass bias although with negative slope. QTOF and LCQ Deca datasets have been shown as examples.
(TIF)

Table S1 Spectra and peptide identifications from concatenated and separate database searches for the standard mix data sets.
(DOC)

Table S2 Spectra and peptide identifications from separate and concatenated database searches for the E. coli and Yeast data sets.
(DOC)
