# Supplementary figures and images for: Learning from Decoys to Improve the Sensitivity and Specificity of Proteomics Database Search Results
Source: PLoS One. 2012 Nov 26;7(11):e50651. doi: 10.1371/journal.pone.0050651 (PMC3506577; doi:10.1371/journal.pone.0050651)

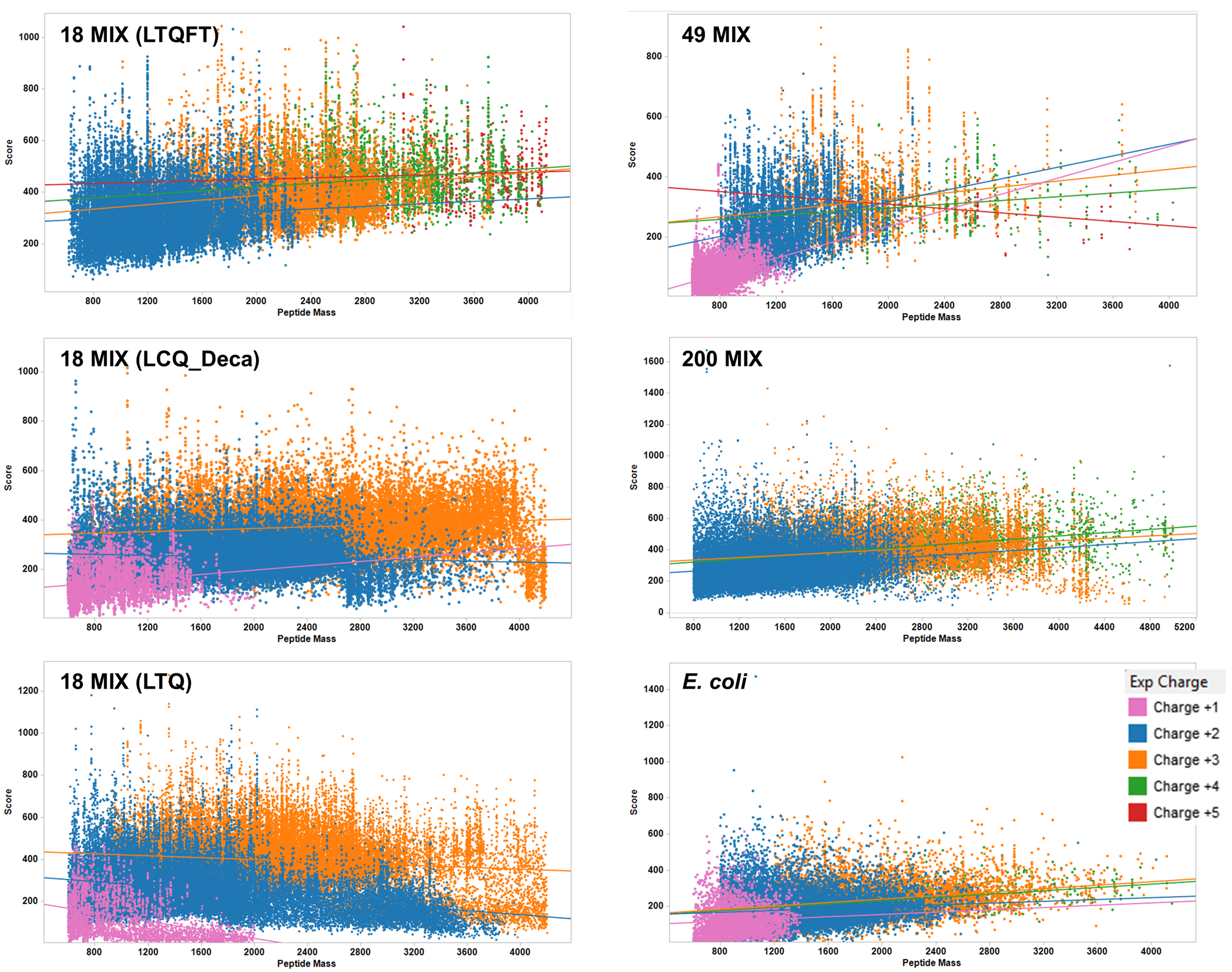

Supplement: Figure S1 — Mass bias trends for few more datasets. The figure shows the mass bias trend as shown for QTOF data in figure 1. This depicts the observation of the mass bias trend for different charge states in few more datasets. This observation is repeatable and forms the basis of FlexiFDR. (TIF) [file pone.0050651.s001.tif]

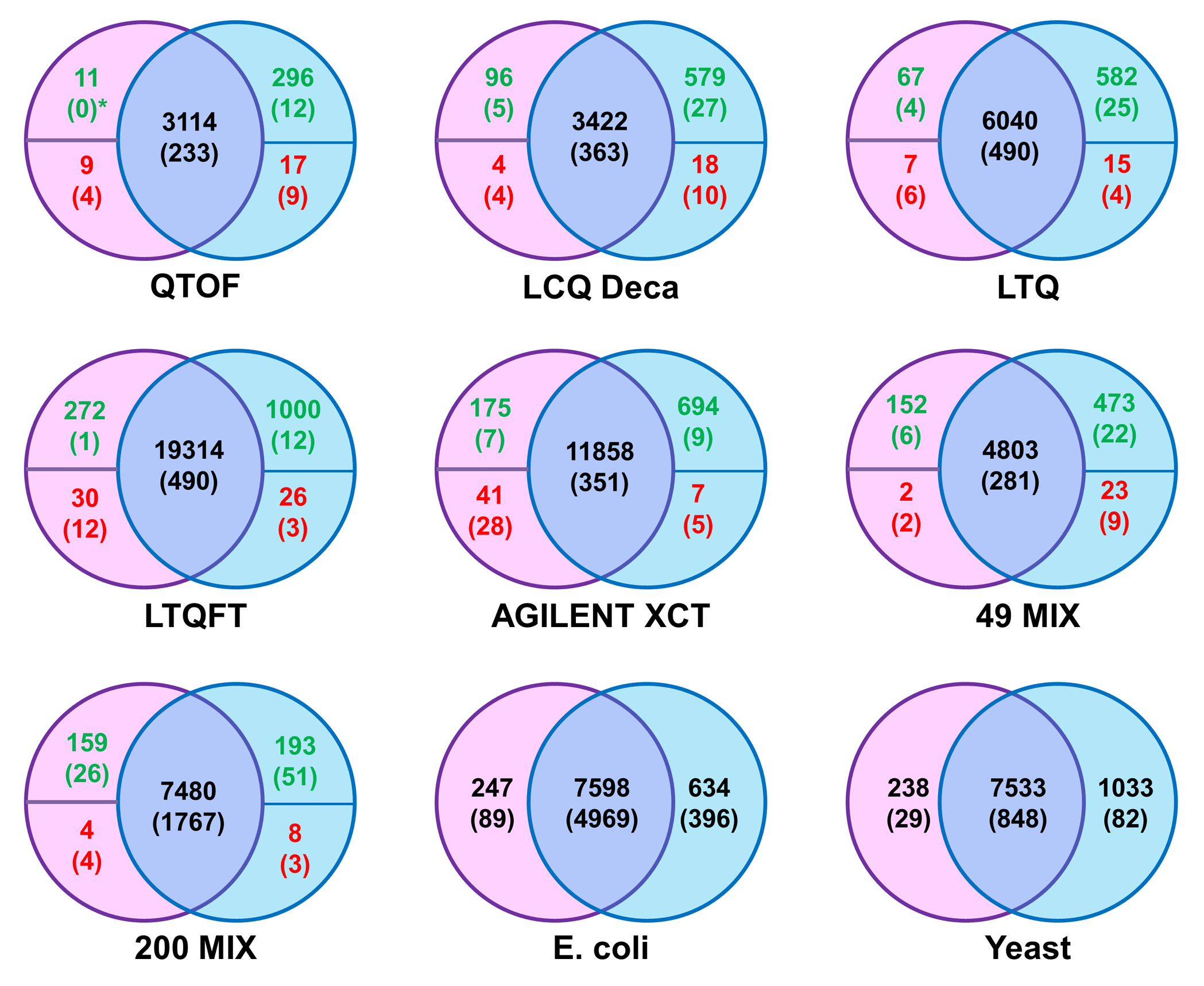

Supplement: Figure S2 — Comparison of spectra and peptides assigned by FDR and FlexiFDR for separate search. Comparison of spectra and peptides assigned by FDR (pink) and FlexiFDR (blue) for separate database search. The number of spectra is shown on top with the number peptides in brackets beneath them. For the standard mixtures, the true positives (green) and false positives (red) identified exclusively are highlighted. FlexiFDR identifies a higher number of true unique spectra and peptides than FDR in almost all cases. The proportion of false positives in exclusively identified set is higher in FDR than FlexiFDR. A star symbol (*) depicts that although there are non-zero true positive spectra identifications in few cases of FDR, they could not bring in any new peptide identification. The peptides they identified were already identified by other spectra (which are shared by both FDR and FlexiFDR). (TIF) [file pone.0050651.s002.tif]

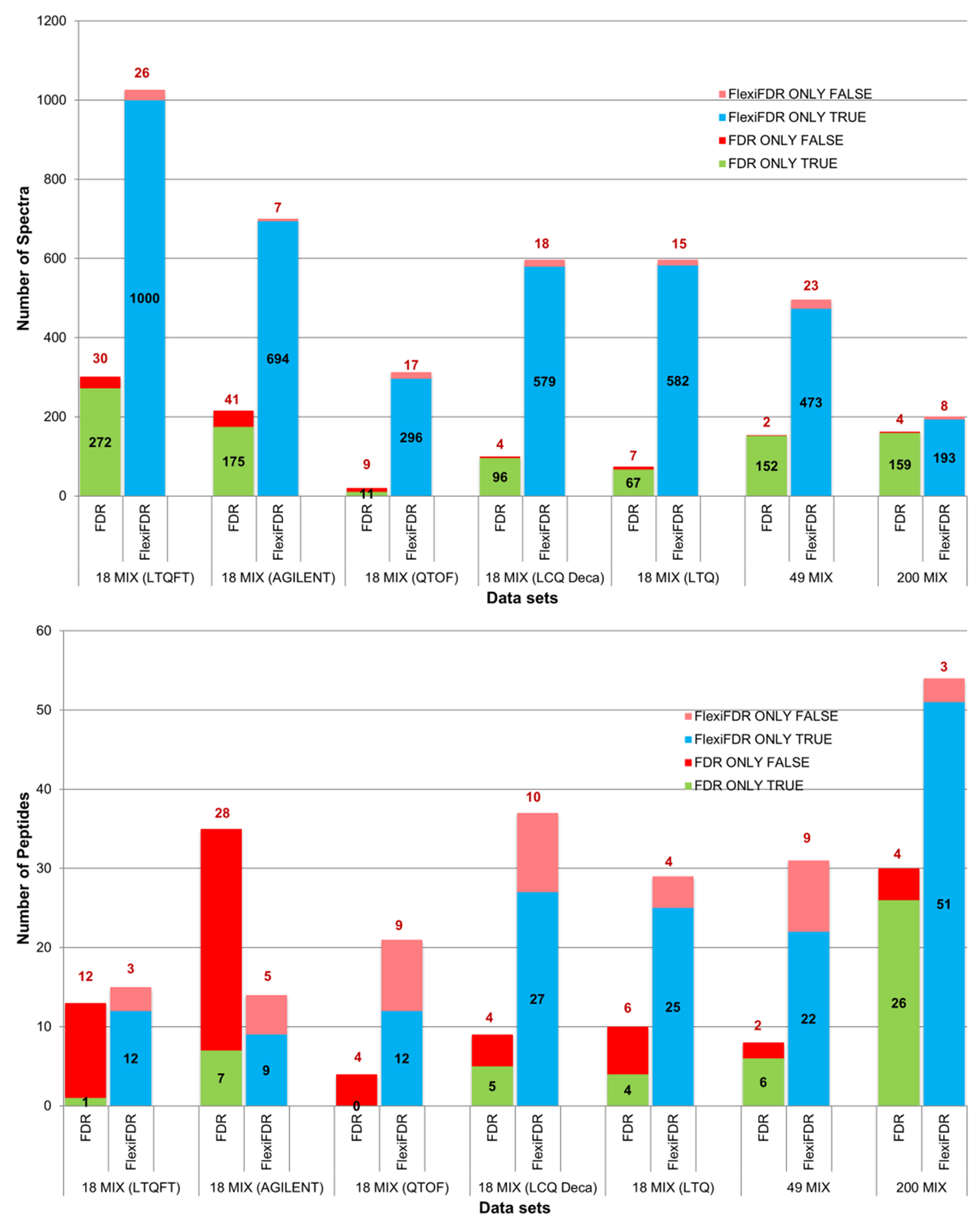

Supplement: Figure S3 — Comparison of unique identifications (spectra and peptides) from separate search. Top and Bottom panels depict spectra and corresponding peptide comparison from a separate search. The blue colored bars represent the unique true hits added by FlexiFDR alone while green colored bars represent unique true hits from FDR alone. Similarly, the pink bars denote false hits from FlexiFDR alone while red bars denote false hits from FDR alone. The spectral hits from FDR can be mapped to unique peptides right in the lower panel. The false spectral hits in case of FDR alone bring more false peptide identifications than FlexiFDR (compare bars from A to B vertically). FlexiFDR brings more unique true hits than FDR and brings lesser number of unique false hits. This enhances the true positives and decreases false positives in the datasets shown. The FlexiFDR method is not search strategy dependent. (TIF) [file pone.0050651.s003.tif]

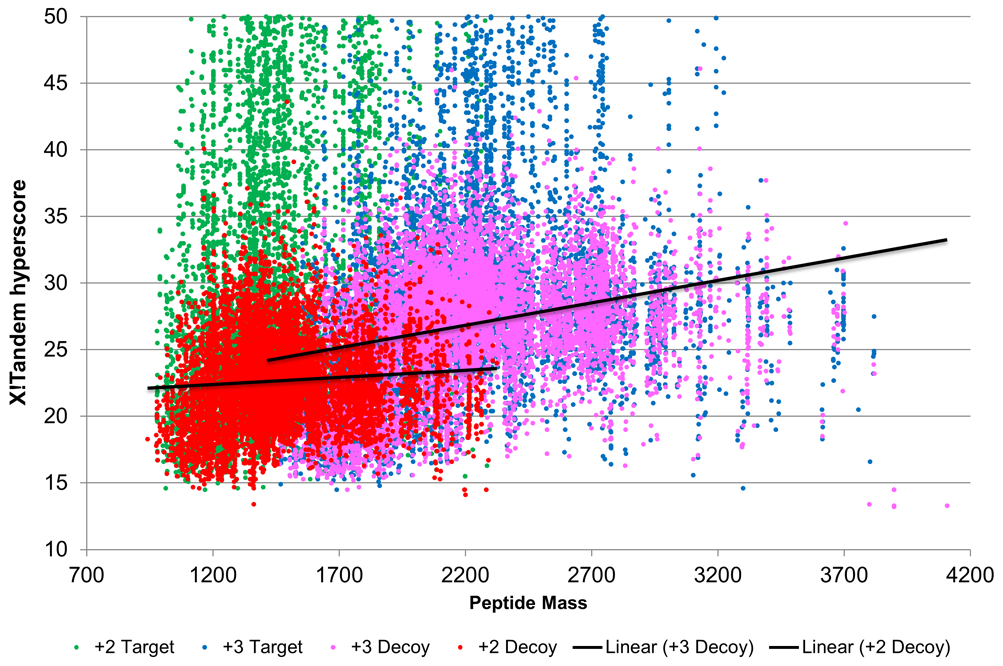

Supplement: Figure S4 — Mass bias trend for X!Tandem hyperscore. Although X!Tandem values do not show mass bias, the hyperscore does show dependence on mass and thus the same trend as MassWiz. QTOF dataset is shown here as an example. (TIF) [file pone.0050651.s004.tif]

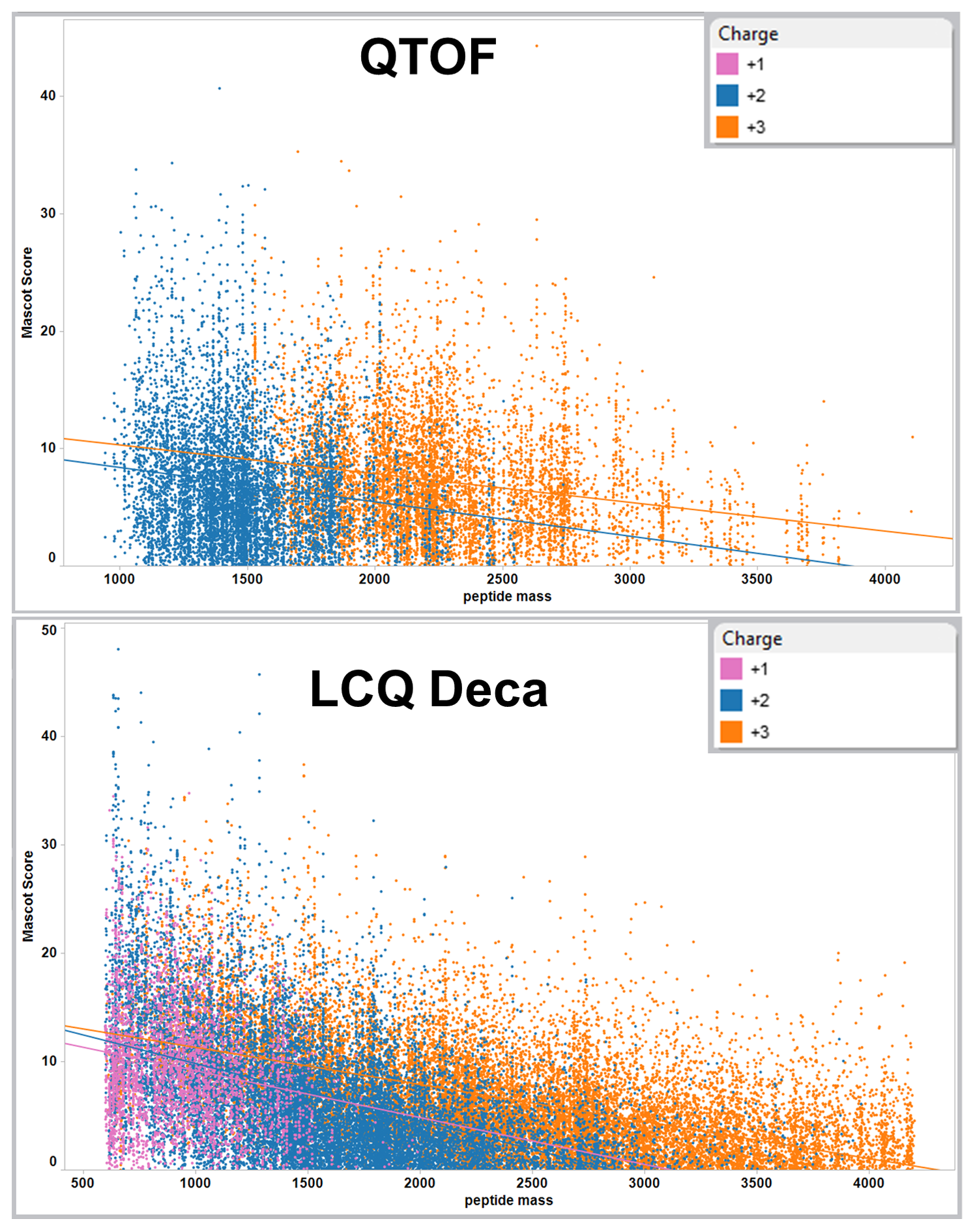

Supplement: Figure S5 — Mass bias trend for Mascot ion score. Mascot ion scores also show a mass bias although with negative slope. QTOF and LCQ Deca datasets have been shown as examples. (TIF) [file pone.0050651.s005.tif]
