# Supplementary material for: Learning from Decoys to Improve the Sensitivity and Specificity of Proteomics Database Search Results
Source: PLoS One. 2012 Nov 26;7(11):e50651. doi: 10.1371/journal.pone.0050651 (PMC3506577; doi:10.1371/journal.pone.0050651)
Supplement: Table S1 — Spectra and peptide identifications from concatenated and separate database searches for the standard mix data sets. (DOC) [file pone.0050651.s006.doc]

**Supporting Table S1: Spectra and peptide identifications from concatenated and separate database searches for the standard mix data sets.**

| Concatenated Search | | | | | | | | | | | | | | | | | |
| --- | --- | --- | --- | --- | --- | --- | --- | --- | --- | --- | --- | --- | --- | --- | --- | --- | --- |
| Dataset | Method | ALL | | | | | | UNIQUE | | | | | | | | | |
|  |  | SPECTRA | | | PEPTIDES | | | SPECTRA | | | | PEPTIDES | | | | | |
|  |  | TRUE | FALSE | NPG (%) | TRUE | FALSE | NPG (%) | TRUE | FALSE | NPG (%) | | TRUE | | FALSE | | NPG (%) | |
| 18 MIX (LTQFT) | FDR | 20510 | 110 |  | 494 | 39 |  | 216 | 44 |  | | 2 | | 12 | |  | |
|  | FlexiFDR | 21588 | 188 | 4.85 | 509 | 42 | 2.25 | 1294 | 122 | 384.62 | | 17 | | 15 | | 85.71 | |
| 18 MIX (AGILENT) | FDR | 12285 | 119 |  | 340 | 76 |  | 56 | 38 |  | | 3 | | 26 | |  | |
|  | FlexiFDR | 12912 | 97 | 5.23 | 345 | 60 | 5.05 | 683 | 16 | 690.43 | | 8 | | 10 | | 72.41 | |
| 18 MIX (QTOF) | FDR | 3156 | 30 |  | 228 | 12 |  | 32 | 14 |  | | 0 | | 7 | |  | |
|  | FlexiFDR | 3558 | 61 | 11.64 | 244 | 26 | 0.83 | 434 | 45 | 806.52 | | 16 | | 21 | | 28.57 | |
| 18 MIX (LCQ Deca) | FDR | 3487 | 5 |  | 363 | 5 |  | 71 | 3 |  | | 5 | | 3 | |  | |
|  | FlexiFDR | 4019 | 20 | 14.81 | 388 | 13 | 4.62 | 603 | 18 | 698.65 | | 30 | | 11 | | 212.50 | |
| 18 MIX (LTQ) | FDR | 6239 | 26 |  | 492 | 16 |  | 130 | 12 |  | | 6 | | 7 | |  | |
|  | FlexiFDR | 6620 | 27 | 6.07 | 506 | 12 | 3.54 | 511 | 13 | 267.61 | | 20 | | 3 | | 138.46 | |
| 49 MIX | FDR | 5841 | 94 |  | 323 | 32 |  | 158 | 28 |  | | 4 | | 11 | |  | |
|  | FlexiFDR | 5883 | 83 | 0.89 | 333 | 29 | 3.66 | 200 | 17 | 28.49 | | 14 | | 8 | | 86.67 | |
| 200 MIX | FDR | 7675 | 26 |  | 1796 | 14 |  | 113 | 5 |  | | 19 | | 4 | |  | |
|  | FlexiFDR | 8063 | 38 | 4.88 | 1886 | 19 | 4.70 | 501 | 17 | 318.64 | | 109 | | 9 | | 369.57 | |
| Separate Search | | | | | | | | | | | | | | | | | |
| Dataset | Method | ALL | | | | | | UNIQUE | | | | | | | | | |
|  |  | SPECTRA | | | PEPTIDES | | | SPECTRA | | | | | PEPTIDES | | | | |
|  |  | TRUE | FALSE | NPG (%) | TRUE | FALSE | NPG (%) | TRUE | FALSE | | NPG (%) | | TRUE | | FALSE | | NPG (%) |
| 18 MIX (LTQFT) | FDR | 20126 | 56 |  | 481 | 23 |  | 272 | 30 | |  | | 1 | | 12 | |  |
|  | FlexiFDR | 20288 | 52 | 0.82 | 491 | 14 | 3.77 | 1000 | 26 | | 242.38 | | 12 | | 3 | | 153.85 |
| 18 MIX (AGILENT) | FDR | 12675 | 83 |  | 333 | 54 |  | 175 | 41 | |  | | 7 | | 28 | |  |
|  | FlexiFDR | 12510 | 49 | -1.03 | 334 | 31 | 6.20 | 694 | 7 | | 256.02 | | 9 | | 5 | | 71.43 |
| 18 MIX (QTOF) | FDR | 3297 | 27 |  | 229 | 9 |  | 11 | 9 | |  | | 0 | | 4 | |  |
|  | FlexiFDR | 3392 | 35 | 2.62 | 240 | 14 | 2.52 | 296 | 17 | | 1385.00 | | 12 | | 9 | | 175.00 |
| 18 MIX (LCQ Deca) | FDR | 3726 | 6 |  | 367 | 6 |  | 96 | 4 | |  | | 5 | | 4 | |  |
|  | FlexiFDR | 3999 | 20 | 6.94 | 388 | 12 | 4.02 | 579 | 18 | | 469.00 | | 27 | | 10 | | 177.78 |
| 18 MIX (LTQ) | FDR | 6445 | 21 |  | 486 | 15 |  | 67 | 7 | |  | | 4 | | 6 | |  |
|  | FlexiFDR | 6608 | 29 | 2.40 | 506 | 13 | 4.39 | 582 | 15 | | 685.14 | | 25 | | 4 | | 230.00 |
| 49 MIX | FDR | 5246 | 5 |  | 286 | 4 |  | 152 | 2 | |  | | 6 | | 2 | |  |
|  | FlexiFDR | 5273 | 26 | 0.11 | 301 | 11 | 2.76 | 473 | 23 | | 194.81 | | 22 | | 9 | | 112.50 |
| 200 MIX | FDR | 7617 | 26 |  | 1782 | 15 |  | 159 | 4 | |  | | 26 | | 4 | |  |
|  | FlexiFDR | 7651 | 30 | 0.39 | 1807 | 14 | 1.45 | 193 | 8 | | 18.40 | | 51 | | 3 | | 86.67 |
|  |  |  |  |  |  |  |  |  |  | |  | |  | |  | |  |
| AVERAGE (SEP + CONCAT) |  |  |  | 4.33 |  |  | 3.55 |  |  | | 460.41 | |  | |  | | 142.94 |

NPG=Net Positive Gain (in %) =100 × (TP+TN-FP-FN)/(FN+TN)

FN+TN=Total Identifications before FlexiFDR was applied, so it was used as reference.
