# Supplementary material for: Learning from Decoys to Improve the Sensitivity and Specificity of Proteomics Database Search Results
Source: PLoS One. 2012 Nov 26;7(11):e50651. doi: 10.1371/journal.pone.0050651 (PMC3506577; doi:10.1371/journal.pone.0050651)
Supplement: Table S2 — Spectra and peptide identifications from separate and concatenated database searches for the E. coli and Yeast data sets. (DOC) [file pone.0050651.s007.doc]

Supporting Table S2: Spectra and peptide identifications from separate and concatenated database searches for the E. coli and Yeast data sets.

| SEPARATE | | | | | | | | | |
| --- | --- | --- | --- | --- | --- | --- | --- | --- | --- |
| Dataset | Method | ALL | | | | UNIQUE | | | |
|  |  | Spectra | Net Gain(%) | Peptide | Net Gain(%) | Spectra | Net Gain(%) | Peptide | Net Gain(%) |
| E coli | FDR | 7845 |  | 5058 |  | 247 |  | 89 |  |
|  | FlexiFDR | 8232 | 4.93 | 5365 | 6.07 | 634 | 156.68 | 396 | 344.94 |
| Yeast | FDR | 7771 |  | 877 |  | 238 |  | 29 |  |
|  | FlexiFDR | 8566 | 10.23 | 930 | 6.04 | 1033 | 334.03 | 82 | 182.76 |
| CONCATENATED | | | | | | | | | |
| Dataset | Method | ALL | | | | UNIQUE | | | |
|  |  | Spectra | Net Gain(%) | Peptide | Net Gain(%) | Spectra | Net Gain(%) | Peptide | Net Gain(%) |
| E coli | FDR | 8445 |  | 5380 |  | 383 |  | 132 |  |
|  | FlexiFDR | 8664 | 2.59 | 5624 | 4.54 | 602 | 57.18 | 376 | 184.85 |
| Yeast | FDR | 7685 |  | 865 |  | 320 |  | 39 |  |
|  | FlexiFDR | 8870 | 15.42 | 965 | 11.56 | 1505 | 370.31 | 139 | 256.41 |
|  |  |  |  |  |  |  |  |  |  |
| **AVERAGE**  **(FDR_s_+FDR_C_)** |  |  | **8.29** |  | **7.05** |  | **229.55** |  | **242.24** |

Net Gain(%)=100 × Number[(FlexiFDR-FDR)/FDR]
